# Tennis vs padel: Precompetitive anxiety as a function of gender and competitive level

**DOI:** 10.3389/fpsyg.2022.1018139

**Published:** 2022-10-06

**Authors:** Alberto Rodríguez-Cayetano, Félix Hernández-Merchán, José Manuel De Mena-Ramos, Antonio Sánchez-Muñoz, Salvador Pérez-Muñoz

**Affiliations:** ^1^Department of Science of Physical Activity and Sport, Pontifical University of Salamanca, Salamanca, Spain; ^2^Department of Science of Physical Activity and Sport, Isabel I University, Burgos, Spain

**Keywords:** sport, racket sports, sport psychology, gender studies, sport performance

## Abstract

The main objective of this research is to analyze the level of anxiety and precompetitive self-confidence of tennis and padel players and to check the influence of gender and competitive level in each of the sports. Four hundred and twenty-three tennis and padel players, with a mean age of 15.40 (± 3.43) years, participated in the study. Of the total number of players surveyed, 291 were padel players and 132 were tennis players. The Spanish version of the Competitive State Anxiety Inventory-2R (CSAI-2R) in the Spanish version was used to measure the level of cognitive anxiety, somatic anxiety and self-confidence between 30 and 35 min before the start of the match. The general results showed that the level of self-confidence of padel players is higher of tennis players. In relation to gender, female tennis players showed a higher level of precompetitive anxiety than male players, while, in padel, it was the opposite. In terms of competitive level, U14 players had the highest level of self-confidence and the lowest level of precompetitive anxiety. When comparing both sports, female tennis players show a higher level of state-anxiety than female padel players and U14 tennis players are the ones who showed a higher level of self-confidence. This study shows how precompetitive anxiety is one of the most important psychological variables in relation to sport performance and how it can vary according to gender and competitive level.

## Introduction

“He is anxious about playing” or “he has anxiety problems” are expressions increasingly used by professionals dedicated to the sports field. The influence of precompetitive anxiety on sport performance has focused the attention of many sport psychologists (Gómez-López et al., 2020), being one of the most studied aspects in sport psychology in recent years (Cuesta-Vargas and Vertedor, 2016; Correia and Rosado, 2019; Pineda-Espejel et al., 2021; Ren et al., 2022).

In this way, competition generates psychological effects on athletes that counteract their psychoemotional resources (Flores et al., 2017; Coco et al., 2020; Jaramillo et al., 2020; Di Corrado et al., 2021). Likewise, one of these main effects is anxiety, which is characterized by an increase in physiological activity that manifests itself in contexts of high emotional and physical demand, accompanied by the activation of the autonomic nervous system (Rodríguez et al., 2015; Castro-Sánchez et al., 2018). In this sense, anxiety is understood as a psycho-emotional negative state of mind characterized by the manifestation of worry and nervousness, finding a cognitive and a somatic component (Weinberg and Gould, 1996). It is regularly associated with restlessness, muscular tension, problems with concentration and fatigue (Ayuso-Moreno et al., 2020; Mehrsafar et al., 2021).

Anxiety is a multidimensional construct in two ways: state anxiety and trait anxiety. State anxiety is understood as an immediate emotional state that appears when the athlete responds with anxiety to specific situations that are characterized by fear, tension and increased physiological arousal and an increase in physiological arousal (Cox, 2012). Trait anxiety is a predisposition to perceive certain environmental situations as threatening and to respond to these situations with increased state anxiety (Spielberger, 1971; Dosil, 2004). This present study focuses on state anxiety.

In addition to state-anxiety and trait-anxiety, the concept of anxiety can be discriminated between the cognitive component and the somatic component, cited above (Andrade et al., 2007). On the one hand, cognitive anxiety manifests with negative thoughts, uneasiness and feelings of insecurity caused by fear of negative social evaluation, failure and loss of self-esteem (Cox, 2012; Rodríguez-Cayetano et al., 2017). On the other hand, somatic anxiety is associated with an increased level of activation of physiological functions produced by nervousness, such as increased heart rate, rapid breathing and muscle tension (Grossbard et al., 2009). Although, it was supposed that these types of anxiety are conceptually unrelated, these two factors are related and interdependant in conditions of stress (Masten et al., 2006) and maintain control over them will make the athlete more likely to succeed in competition (Kotnik et al., 2012).

With respect to sport, the previous moment to competition is the most propitious for anxiety to occur (Dosil, 2004). This type of anxiety is known as precompetitive anxiety (Cox, 2012), being one of the psychological factors that most influence competitive sport performance, along with self-confidence (León-Prados et al., 2014; Pineda-Espejel et al., 2019). Research in the sport context shows how athletes with high levels of anxiety achieved worse results than those who showed lower levels of anxiety in competition (León-Prados et al., 2011; López-Torres et al., 2011; Ngo et al., 2017; Sánchez et al., 2017). On the other hand, the athlete’s self-confidence, understood as the athlete’s belief that he/she can perform successfully in competition (Robazza and Bortoli, 2007), has a positive correlation with sport performance (Santos-Rosa et al., 2007; Díaz et al., 2008; Martínez-Romero et al., 2016; Zurita-Ortega et al., 2017), although an excess of self-confidence can cause a decrease in the optimal level of performance (Weinberg and Gould, 1996). The correlation between self-confidence and competitive performance is one of the most important aspects of competitive performance for athletes (Vodicar et al., 2012).

The dimensions of competitive anxiety tend to be influenced by numerous factors, such as gender or type of sport (Martens et al., 1990). Racquet sports present a series of physical characteristics and physiological demands different from other types of sports due to the nature of their game (Ford et al., 2017), with intermittent sprints and incomplete recoveries between points (Alvero-Cruz et al., 2005; Courel-Ibáñez et al., 2017; Castillo-Rodríguez et al., 2022) and constant decision making in a very short period of time (Castillo-Rodríguez et al., 2014) and, in turn, being accurate to reduce the inaccuracies that arise during the course of the game (Barahona-Fuentes et al., 2019). In this regard, the ability of the players to deal with pressure situations is directly related to their performance (González-Díaz et al., 2012; Knight et al., 2016; Martínez-Gallego et al., 2022).

Most previous studies that have studied the difference in the level of precompetition anxiety between men and women have shown that women show a higher level of somatic and cognitive anxiety and a lower level of self-confidence than men in the moments prior to competition (Dias et al., 2010; Gutiérrez et al., 2013; Ruiz-Juan and Zarauz, 2013; Ramis et al., 2015). However, Zarauz-Sancho et al. (2016) found higher levels of somatic anxiety in male runners than in female runners.

In the sport of tennis, several studies have shown that female tennis players showed a higher level of cognitive and somatic anxiety and a lower level of self-confidence than men before the sport competition (Correia and Rosado, 2019; Khot and Bujurke, 2021; Martínez-Gallego et al., 2022). In addition, other studies have shown that anxiety affects female professional tennis players more at key moments of the match and the tournament prize money (Cohen-Zada et al., 2017). However, Keskin et al. (2021) found no significant differences in the level of cognitive anxiety and somatic anxiety in adult tennis players in the moments before the competition and, even, that women showed a lower level of precompetitive anxiety than men with increasing age (Ebbeck, 1994). With respect to padel, few studies have analyzed the level of precompetitive anxiety in this sport (Almendros-Pacheco et al., 2022; Castillo-Rodríguez et al., 2022). It is worth highlighting the study by Castillo-Rodríguez et al. (2022) in which they showed that the level of self-confidence of the players increased as the player competed in a higher category.

For this reason, the literature shows the need to analyze and work on the psychological aspect in order to reduce the level of anxiety of each athlete and, thus, achieve a better sporting performance. For this reason, the main objective of this research is to analyze the level of anxiety and precompetitive self-confidence of tennis and padel players and to check the influence of gender and competitive level in each of the sports.

With reference to this main objective, it was hypothesized that:

1. Tennis players would have higher values of cognitive and somatic anxiety and lower values of self-confidence than padel players.
2. Female tennis and padel players will have a higher level of cognitive and somatic anxiety and a lower level of self-confidence than male tennis and padel players.
3. U14 players would have a higher level of self-confidence and a lower level of precompetitive anxiety, since, although they are federated and competitive athletes, they are just beginning their competitive stage.
4. Male and female padel players would have a higher level of self-confidence and less precompetitive anxiety than tennis players, as they have the support of a partner in the competition.
5. Padel players would have lower values of precompetitive anxiety and a higher level of self-confidence than tennis players, regardless of their competitive level.
6. Two variables related to anxiety would correlate positively with each other, while both would correlate negatively with self-confidence.

## Materials and methods

### Research design

The study design is cross-sectional descriptive. The type of sampling was non-probabilistic by convenience, i.e., the selection of participants was based on the presence of characteristics that respond to the needs of the research (Otzen and Manterola, 2017). Players were selected who competed in federated tennis and padel tournaments and who trained a minimum of 3 h per week in the selected categories.

### Participants

A sample of 423 tennis and padel players with a mean age of 15.40 (± 3.43) years participated in this study. Of the total number of players surveyed, 291 were padel players and 132 were tennis players. By gender, there were a total of 191 males and 100 female padel players, while, in tennis, there were 85 male and 47 female players. Finally, in terms of competitive level, in padel, there were a total of 93 Under14 (U14) players, 93 Under16 (U16) players and 105 in the senior category, while, in tennis, there were a total of 31 U14 athletes, 34 U16 and 67 players who competed in the senior category.

The sample was obtained from various sports clubs that organized federated tournaments in both sports, requesting the voluntary participation of the athletes. All players were informed about the characteristics and objectives of the study and signed an informed consent form. Finally, the non-repetition of individuals was guaranteed with individualized follow-up during data collection to avoid duplication of data during the selection process.

### Instruments

The Spanish version of the Competitive State Anxiety Inventory-2R (CSAI-2R) by Cox et al. (2003) in the Spanish version by Andrade et al. (2007) was used to measure the level of precompetitive anxiety of tennis and padel players.

This instrument is composed of 16 items using a Likert-type response format, with four different alternatives, numbered from 1 (not at all) to 4 (very much). These items make up a total of three subscales: cognitive anxiety, somatic anxiety and self-confidence.

An analysis of the instrument indicated that the reliability measured by Cronbach’s alpha was 0.825 for cognitive anxiety, 0.778 for somatic anxiety and 0.771 for self-confidence, which is considered very good internal consistency (Nunnally and Bernstein, 1994; Vaughn et al., 2012).

### Procedure

The study was consistent with the Helsinki Declaration of 2013. Participants were treated ethically under the American Psychological Association code of ethics regarding consent, anonymity and responses. Also, the study is covered by the current Spanish legal regulations governing research on human subjects (RD 561/1993), respecting at all times privacy and the law on the protection of personal data (Organic Law 15/1999).

Firstly, the people in charge of organizing the tournaments were contacted to request permission to administer the questionnaires to the players before the start of the competition. In order to respect the principle of voluntariness and confidentiality, each player signed an informed consent form (in the case of minors, their legal representatives did so), in which the objectives of the research and their voluntary participation in it were detailed.

The questionnaire was administered to the players between 30 and 45 min before the start of each of the matches in all cases, following the same criteria as Andrade et al. (2007) in which they administered the questionnaire between 15 and 45 min before the start of the competition. Finally, it is essential to highlight that the questionnaire was administered in tennis in individual matches (1vs1) while padel matches were played in their usual format (2vs2).

### Statistical analysis

All data were analyzed using the statistical package SPSS for Windows v.25.0 (*SPSS Inc.,* Chicago, IL, United States). Descriptive analysis was initially conducted. The test is considered a valid statistical procedure when skewness and kurtosis range between −1 and 1 (Blanca et al., 2017; Fernández-Río et al., 2022) and, in the present study, these were (Sk = 0.357; Ku = −0.730) for cognitive anxiety, (Sk = 0.873; Ku = 0.230) for somatic anxiety and (Sk = −0.627; Ku = 0.403) for self-confidence.

To analyze the differences between the anxiety factors with the independent variables sport (tennis/padel), gender (male/female) and competitive level (U14, 16 and Senior), a one-way ANOVA test was performed for each of them. Significant interactions were calculated using *post hoc* tests with Bonferroni test, using a level of significance of 0.05. The effect size was assessed using Cohen’s *d* test (Cohen, 1988). The threshold values for the Cohen effects sizes in the ANOVA test were small: 0.10; moderate, 0.25; and large, 0.40. Finally, Pearson’s correlation coefficient was used to analyze the correlations between the psychological variables at a general level and according to the sport practiced.

## Results

Firstly, the overall results obtained among tennis and padel players showed a higher level of self-confidence, with significant differences [*f*_(1.00)_ = 4.51; d = 0.223; *p* < 0.05], than cognitive anxiety and somatic anxiety, the latter being the one with the lowest values (Table 1).

**Table 1:** Precompetitive anxiety as a function of the practiced sport modality.

	Padel	Tennis	Value of *p*	*f*	*d* Cohen
CA	2.01 (± 0.679)	2.07 (± 0.781)	0.421	0.65	0.079
SA	1.60 (± 0.557)	1.50 (± 0.467)	0.055	3.71	0.202
SC	3.25 (0.548)	3.13 (0.548)	0.034^*^	4.51	0.223

* *p* < 0.05.

CA, Cognitive anxiety; SA, Somatic anxiety; SC, Self-confidence.

Regarding the gender of the players and the sport played, male padel players obtained higher values in the three factors analyzed than female, highlighting significant differences in the somatic anxiety variable (*f*_(1.00)_ = 14.49; d = 0.459; *p* < 0.001). With respect to tennis, female players showed higher values in the variables related to anxiety, with significant differences in cognitive anxiety (*f*_(1.00)_ = 8.40; d = 0.527; *p* < 0.001) and lower self-confidence, without significant differences, in the moments prior to the start of the competition (Table 2).

**Table 2:** Precompetitive anxiety in racket sport players as a function of gender.

	Padel					Tennis				
	Girls	Boys	Value of *p*	*f*	*d* Cohen	Girls	Boys	Value of *p*	*f*	*d* Cohen
CA	1.96 (0.67)	2.04 (0.68)	0.363	0.83	0.118	2.33 (0.59)	1.93 (0.84)	0.004^*^	8.40	0.527
SA	1.44 (0.52)	1.69 (0.56)	0.000^**^	14.49	0.459	1.56 (0.53)	1.46 (0.43)	0.280	1.18	0.214
SC	3.22 (0.56)	3.27 (0.54)	0.497	0.46	0.073	3.00 (0.52)	3.19 (0.65)	0.088	2.95	0.315

* *p* < 0.05;

** *p* < 0.001.

Referring to the competitive level, senior padel players showed the highest values in cognitive anxiety and somatic anxiety and the lowest level of self-confidence, with significant differences in all factors (*p* < 0.001). On the other hand, in tennis, U16 players showed the highest level in factors related to precompetitive anxiety and the lowest level of self-confidence, with significant differences in all of them (*p* < 0.001) (Table 3).

**Table 3:** Precompetitive anxiety in racket sport players as a function of competitive level.

	Padel					Tennis				
	U14	U16	Senior	Value of *p*	*f*	U14	U16	Senior	Value of *p*	*f*
CA	1.79 (0.70)^3^	1.96 (0.55)	2.26 (0.69)^1^	0.000^**^	13.39	1.13 (0.29)^2^^,^^3^	2.56 (0.42)^1^^,^^3^	2.26 (0.71)^1^^,^^2^	0.000^**^	57.61
SA	1.44 (0.55)^3^	1.52 (0.51)^3^	1.82 (0.54)^1^^,^^2^	0.000^**^	14.31	1.09 (0.15)^2^^,^^3^	1.71 (0.54)^1^	1.58 (0.41)^1^	0.000^**^	21.71
SC	3.44 (0.54)^3^	3.27 (0.49)^3^	3.07 (0.55)^1^^,^^2^	0.000^**^	12.94	3.69 (0.40)^2^^,^^3^	2.83 (0.43)^1^	3.01 (0.60)^1^	0.000^**^	25.30

** *p* < 0.001. Bonferroni *Post-hoc* test are given below.

1 Differences with U14.

2 Differences with U16.

3 Differences with Senior; CA, Cognitive anxiety; SA, Somatic anxiety; SC, Self-confidence.

**Figure 1:**
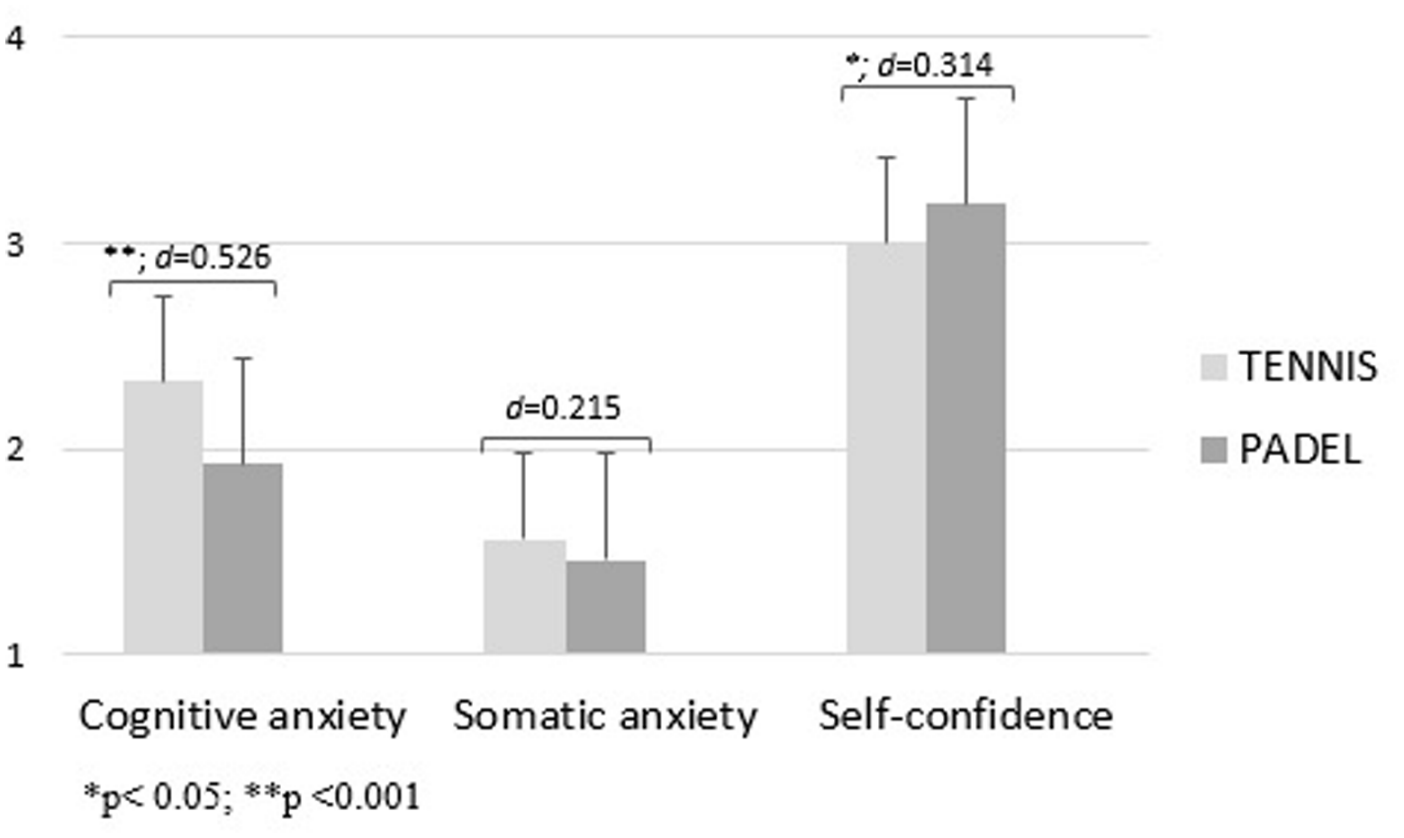
Precompetitive anxiety in female racket sports players as a function of the sport played.

**Figure 2:**
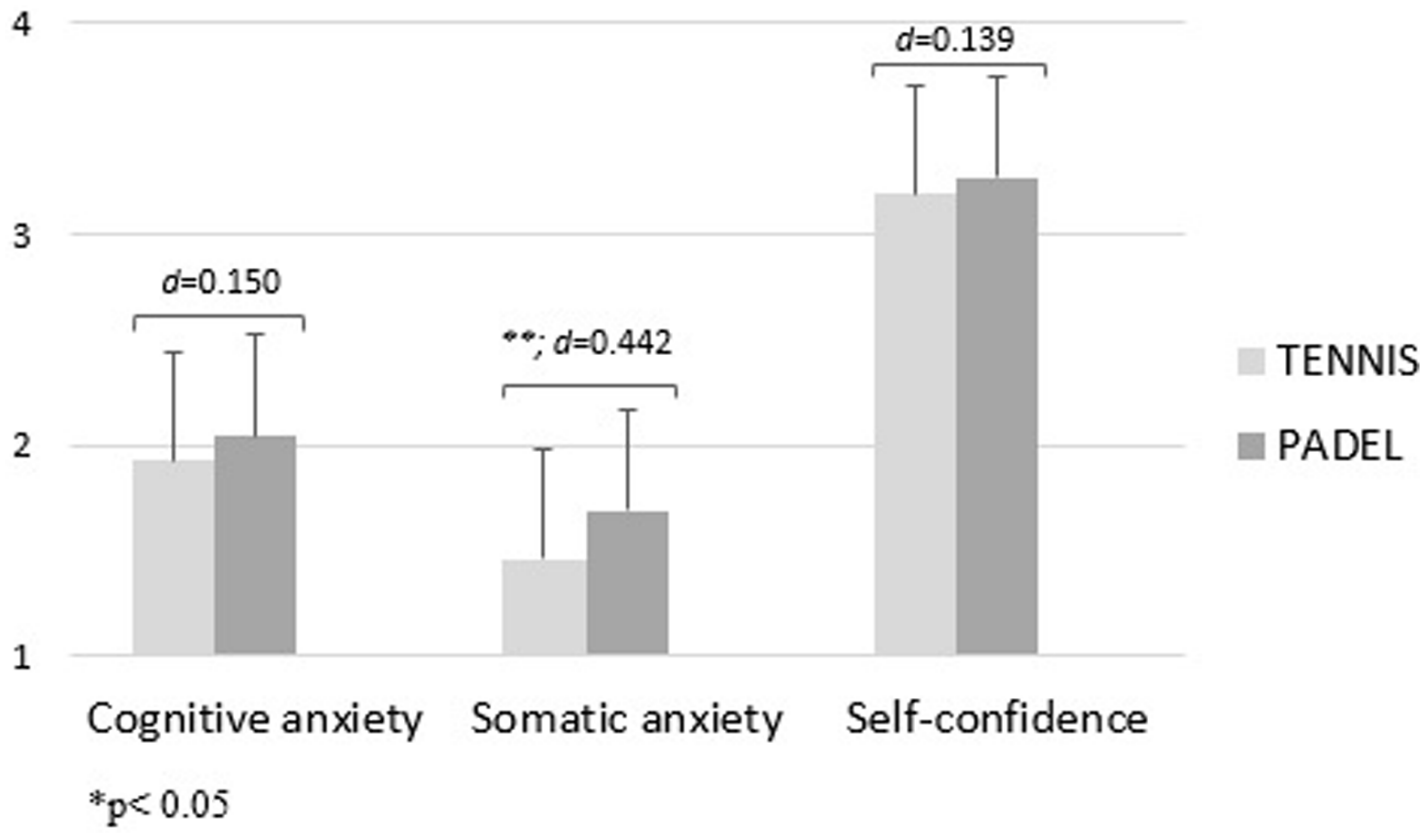
Precompetitive anxiety in male racket sports players as a function of the sport played.

Figures 1, 2 show the level of precompetitive anxiety of female and male racket sports players as a function of the sport played. Female tennis players showed a significantly higher level of cognitive anxiety [*f*_(1.00)_ = 10.34; d = 0.526; *p* < 0.001] and a significantly lower level of self-confidence [*f*_(1.00)_ = 5.05; d = 0.314; *p* < 0.05] than female padel players. For boys, padel players obtained higher values in all three variables than tennis players, showing significant differences in the somatic anxiety factor [*f*_(1.00)_ = 11.30; d = 0.442; *p* < 0.001; Figures 1, 2].

**Figure 3:**
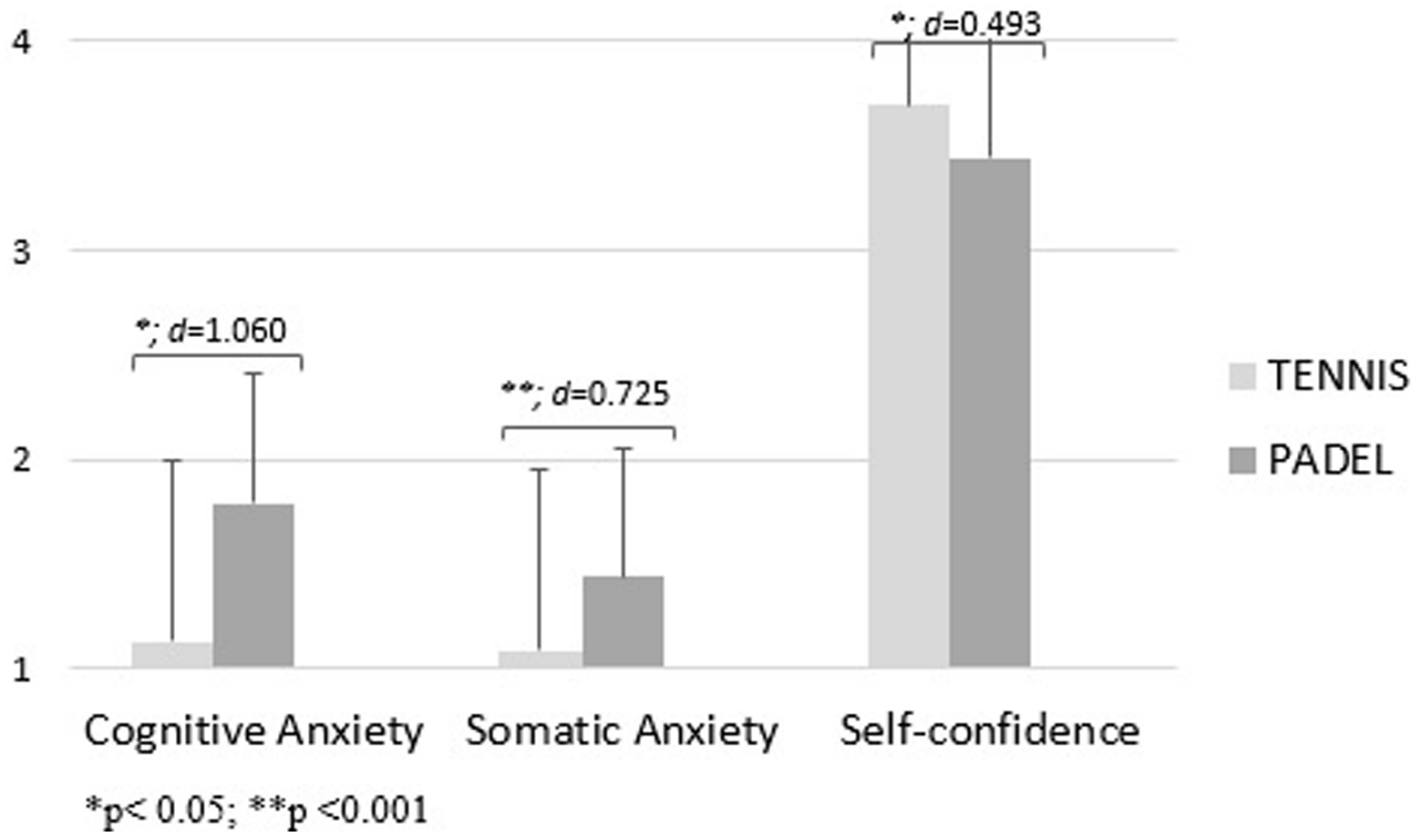
Precompetitive anxiety in U14 racket sports players as a function of the sport played.

**Figure 4:**
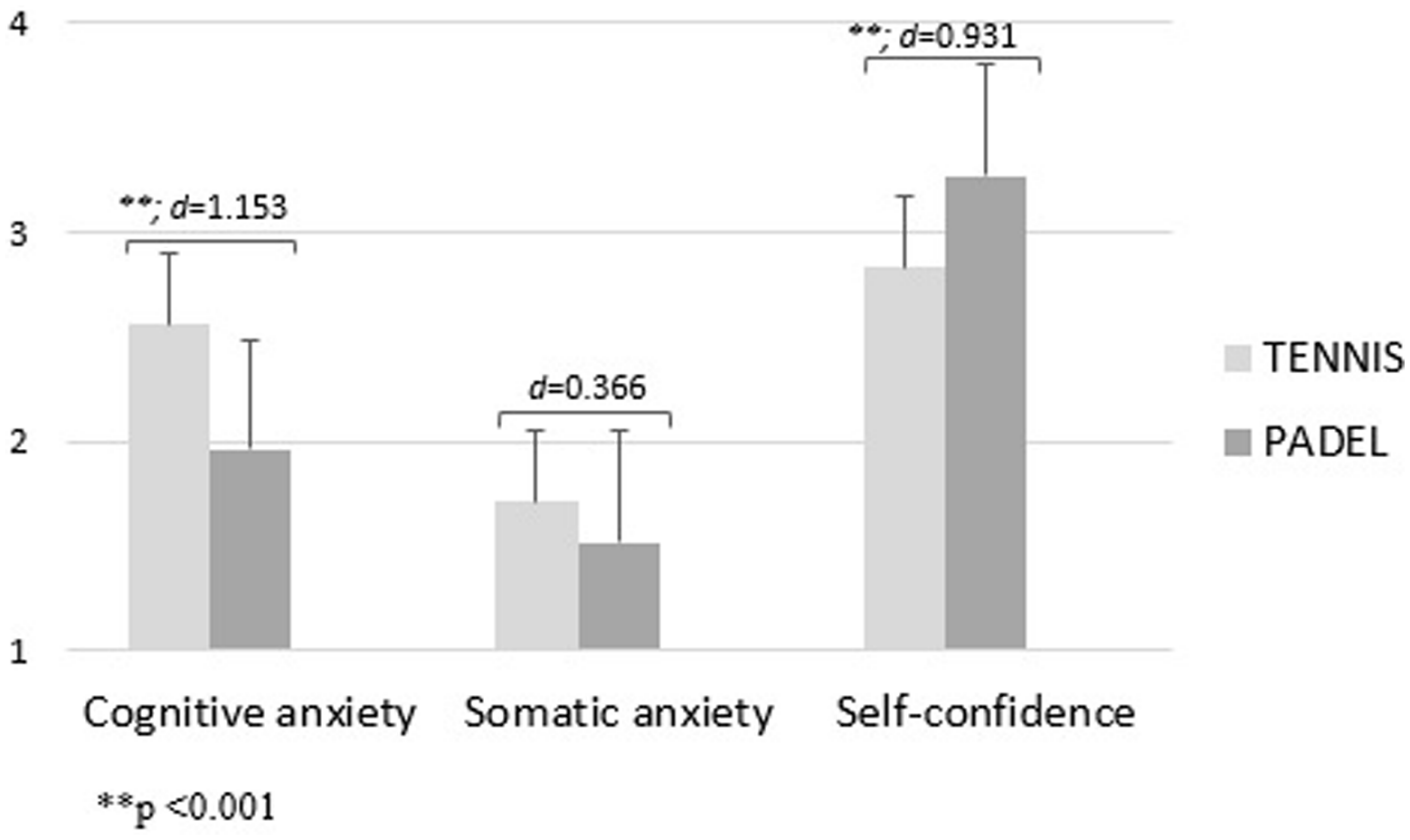
Precompetitive anxiety in U16 racket sports players as a function of the sport played.

**Figure 5:**
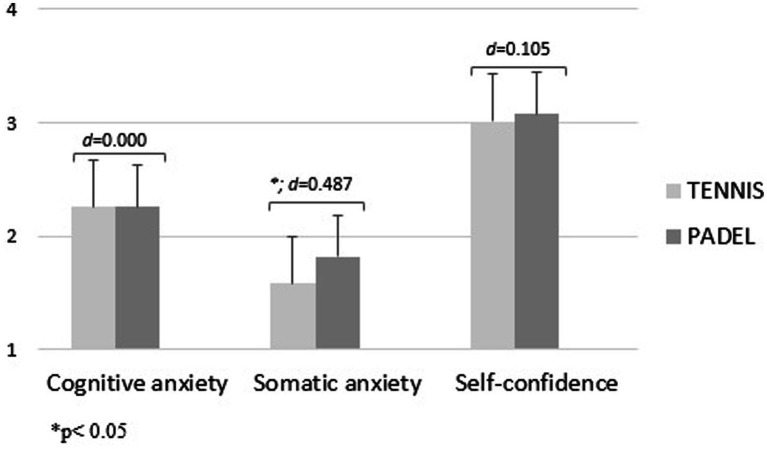
Precompetitive anxiety in senior racket sports players as a function of the sport played.

In addition, Figures 3–5 show the level of precompetitive anxiety of racket sports players as a function of competitive level and sport played. U14 padel players showed higher values of cognitive anxiety [*f*_(1.00)_ = 25.80; d = 1.060; *p* < 0.05] and somatic anxiety [*f*_(1.00)_ = 12.67; d = 0.725; *p* < 0.001] and a lower level of self-confidence than tennis players [*f*_(1.00)_ = 5.42; d = 0.493; *p* < 0.05; Figure 3]. In contrast, U16 tennis players showed a higher level of cognitive anxiety [*f*_(1.00)_ = 33.26; d = 1.153; *p* < 0.001] and somatic anxiety and a lower level of self-confidence [*f*_(1.00)_ = 21.75; d = 0.931; *p* < 0.001; Figure 4]. Finally, for senior players, padel players showed a higher level of somatic anxiety than tennis players [*f*_(1.00)_ = 9.84; d = 0.487; *p* < 0.005; Figure 5].

Finally, bivariate correlations are established between the variables of precompetitive anxiety at a general level and according to the sport practiced. As can be seen, in all cases, there is a significant positive correlation between cognitive anxiety and somatic anxiety and both correlate significantly negatively with self-confidence (Table 4).

**Table 4:** Correlation coefficient between precompetitive anxiety variables and sport practiced.

	SA	SC
**Racket sport players**		
CA	0.522^**^	−0.415^**^
SA		−0.317^**^
**Tennis players**		
CA	0.641^**^	−0.591^**^
SA		−0.521^**^
**Padel players**		
CA	0.487^**^	−0.312^**^
SA		−0.254^**^

** *p* < 0.01.

CA, cognitive anxiety; SA, somatic anxiety; SC, self-confidence.

## Discussion

The main objective of this research was to analyze the level of precompetitive anxiety and self-confidence of tennis and padel players and to test the influence of gender and competitive level in each sport. As an initial hypothesis, it was proposed that tennis players would have higher values of cognitive and somatic anxiety and lower values of self-confidence than padel players. This hypothesis was partially accepted, since, as the results indicate, tennis players showed a significantly lower level of self-confidence than padel players. There are no previous studies comparing the level of precompetitive anxiety and self-confidence between tennis and padel players with similar characteristics, but these results can be related to previous studies that showed that team sports players showed a higher level of self-confidence than individual sports players and a higher level of cognitive anxiety (Dias et al., 2010; Correia and Rosado, 2019; Marín-González et al., 2022). However, they do not follow the line of other research in which no differences were found between the type of sport played, individual or team (Hanton et al., 2008; O’Donoghue and Neil, 2015). However, the results obtained may be due to the fact that in tennis, being an individual sport, there is a greater concern about not performing at the highest level, which leads to a decrease in self-confidence, as there is no one who can cooperate with you to achieve the goal of victory.

As a second hypothesis, it was established that female tennis and padel players will have a higher level of cognitive and somatic anxiety and a lower level of self-confidence than male tennis and padel players. This hypothesis was only partially valid, since, in tennis, female tennis players showed lower values in self-confidence and higher values in somatic anxiety and cognitive anxiety, with significant differences in the latter. These results are in line with the study conducted by Martínez-Gallego et al. (2022) with 42 male and female tennis players between 12 and 18 years old, Filaire et al. (2009) and Keskin et al. (2021) with adult tennis players and Wang (2021) with 187 table tennis players in which they showed differences in anxiety-related variables and lower values in self-confidence, although there were no significant differences. Furthermore, they also corroborate the line of Di Corrado et al. (2021) in which they demonstrated that female athletes showed higher levels of tension, stress and anger than male athletes, key psychological aspects for sport performance. This may be due to the fact that, when facing the challenge of competition alone, it causes doubts in the sporting performance of these players. For these reasons, it is coherent to think about the importance of establishing psychological training with special emphasis on female players in order to improve the mental aspect before the start of the competition. It is important to find their own identity which should be independent of stereotypes (Masten et al., 2006). On the contrary, in padel, it is the players who showed the highest values in the three variables analyzed, showing significant differences in somatic anxiety, so the hypothesis initially put forward for this sport is not fulfilled. These results do not follow the line of Rodríguez-Cayetano et al. (2017) who, despite not finding significant differences in any of the three factors, padel players showed a higher level of self-confidence and a lower level of cognitive anxiety. In this sport, it may be advisable to establish guidelines and routines to work on cohesion between the couple to improve the level of self-confidence prior to the competition.

The third hypothesis was related to the competitive level of the players. It was established that U14 players would have a higher level of self-confidence and a lower level of precompetitive anxiety, since, although they are federated and competitive athletes, they are just beginning their competitive stage. In both sports, this hypothesis was completely fulfilled, as it was the U14 players who obtained lower values of precompetitive anxiety and a higher level of self-confidence, with significant differences in all factors. These results are in line with other studies related to racket sports for these ages (Martínez-Gallego et al., 2022). In contrast, in the study conducted by Castillo-Rodríguez et al. (2022) with 100 padel players, the players who competed at a higher competitive level were the ones who obtained a higher level of self-confidence, unlike in this research where the senior players obtained the lowest level of self-confidence and higher values of anxiety. This may be due to the fact that these players are the ones who seek a higher sporting performance and are focused on the pursuit of a result in the competition, which makes them approach the sporting event with a higher level of stress. However, it is important to note that in tennis, it was the U16s who showed the highest levels of anxiety and the lowest levels of self-confidence. This may be explained by the adolescents’ desire to achieve victory in competition, compared to children who may still see sport as a game (Crocker and Park, 2004).

In relation to the fourth hypothesis, it was established that male and female padel players would have a higher level of self-confidence and less precompetitive anxiety than tennis players, as they have the support of a partner in the competition. This hypothesis was fulfilled in the female modality, as female tennis players obtained a significantly higher level of cognitive anxiety and a significantly lower level of self-confidence. This fact may be one of the reasons for the high female participation in this sport nowadays, as more than 30% of the existing licenses in Spain, one of the countries with the highest number of professional padel athletes, are girls (Courel-Ibáñez et al., 2017). On the other hand, in the male modality this is not the case, as the padel players are the ones who have shown the highest values in the three variables, with significant differences in the somatic anxiety variable. It should be noted that the players of both sports have relatively low anxiety values and a high level of self-confidence, which may be due to the fact that, by playing sports in which there is constant decision making and acceptance of making mistakes on a constant basis, they are athletes used to withstanding a high level of mental pressure both in the competition and in the moments prior to it (González-Díaz et al., 2012; Knight et al., 2016).

The fifth hypothesis was that padel players would have lower values of precompetitive anxiety and a higher level of self-confidence than tennis players, regardless of their competitive level. This hypothesis was fulfilled with the U16 players, as the tennis players showed a significantly higher level of cognitive anxiety and a significantly lower level of self-confidence. On the other hand, with U14 players it was not fulfilled, as U14 tennis players are the ones who showed a lower level of anxiety and a higher level of self-confidence of all the categories analyzed, with significant differences in all three variables. Moreover, this was not true for senior players either, as padel players showed significantly higher values in somatic anxiety. There are no previous studies that have compared the level of precompetitive anxiety between tennis and padel players according to the competitive level, but it can be affirmed that specific training programs should be established at a mental level to maintain over time the levels obtained in these three variables in children’s tennis players with the aim of learning skills to improve future sporting performance as the competitive level increases.

Finally, it was hypothesized that the two variables related to anxiety would correlate positively with each other, while both would correlate negatively with self-confidence. The results obtained showed that this hypothesis was completely fulfilled, since, both at a general level and for each of the sport modalities, this statement was fulfilled. These results show the importance of working on players’ self-confidence to improve their sporting performance (Martínez-Romero et al., 2016; Zurita-Ortega et al., 2017).

This study has several strengths. Firstly, it is the first research that compares the level of precompetitive anxiety in tennis and padel tennis players, the two most played racket sports in the world today. Secondly, the results obtained have a very important practical character for coaches and sport psychologists, especially taking into account the differences by gender and competitive level. The results should be taken into account in order to implement appropriate training programs for each individual athlete, both on and off the track.

Although this study follows a methodology very similar to other recent research carried out in this field, it is important to highlight some limitations of this research work. To measure anxiety, it would be advisable to be able to use not only questionnaires, but also other types of tools that can help to measure the level of precompetitive anxiety and to relate it to other psychological variables that are essential for sports performance. Future studies should relate the level of precompetition anxiety with the player’s performance in the competition and some other post-match psychological characteristics such as, for example, mood, and thus be able to relate these first data obtained by comparing both sports to a greater extent.

## Conclusion

In conclusion, the results of the present study show how tennis and padel players have a higher level of self-confidence than pre-competitive anxiety, as well as the influence of several factors such as gender and competitive level on the psychological characteristics of the athletes prior to the start of the match.

## Data availability statement

The original contributions presented in the study are included in the article/supplementary material, further inquiries can be directed to the corresponding author.

## Ethics statement

Ethical review and approval was not required for the study on human participants in accordance with the local legislation and institutional requirements. Written informed consent to participate in this study was provided by the participants’ or participants’ legal guardian/next of kin.

## Author contributions

AR and SP: conceptualization, methodology, investigation, formal analysis, and writing—original draft. FH and JM: data curation. AR, JM, and SP: performed the statistical analysis. AR: supervision. All authors contributed to the article and approved the submitted version.

## Conflict of interest

The authors declare that the research was conducted in the absence of any commercial or financial relationships that could be construed as a potential conflict of interest.

## Publisher’s note

All claims expressed in this article are solely those of the authors and do not necessarily represent those of their affiliated organizations, or those of the publisher, the editors and the reviewers. Any product that may be evaluated in this article, or claim that may be made by its manufacturer, is not guaranteed or endorsed by the publisher.
